# Diet evolution of carnivorous and herbivorous mammals in Laurasiatheria

**DOI:** 10.1186/s12862-022-02033-6

**Published:** 2022-06-21

**Authors:** Yonghua Wu

**Affiliations:** 1 School of Life Sciences, Northeast Normal University, 5268 Renmin Street, 130024 Changchun, China; 2 Jilin Provincial Key Laboratory of Animal Resource Conservation and Utilization, Northeast Normal University, 2555 Jingyue Street, 130117 Changchun, China

**Keywords:** Dietary evolution, Carnivores, Herbivores, Digestive system genes, Positive selection

## Abstract

**Background:**

Laurasiatheria contains taxa with diverse diets, while the molecular basis and evolutionary history underlying their dietary diversification are less clear.

**Results:**

In this study, we used the recently developed molecular phyloecological approach to examine the adaptive evolution of digestive system-related genes across both carnivorous and herbivorous mammals within Laurasiatheria. Our results show an intensified selection of fat and/or protein utilization across all examined carnivorous lineages, which is consistent with their high-protein and high-fat diets. Intriguingly, for herbivorous lineages (ungulates), which have a high-carbohydrate diet, they show a similar selection pattern as that of carnivorous lineages. Our results suggest that for the ungulates, which have a specialized digestive system, the selection intensity of their digestive system-related genes does not necessarily reflect loads of the nutrient components in their diets but appears to be positively related to the loads of the nutrient components that are capable of being directly utilized by the herbivores themselves. Based on these findings, we reconstructed the dietary evolution within Laurasiatheria, and our results reveal the dominant carnivory during the early diversification of Laurasiatheria. In particular, our results suggest that the ancestral bats and the common ancestor of ruminants and cetaceans may be carnivorous as well. We also found evidence of the convergent evolution of one fat utilization-related gene, *APOB*, across carnivorous taxa.

**Conclusions:**

Our molecular phyloecological results suggest that digestive system-related genes can be used to determine the molecular basis of diet differentiations and to reconstruct ancestral diets.

**Supplementary Information:**

The online version contains supplementary material available at 10.1186/s12862-022-02033-6.

## Background

Laurasiatheria includes typical carnivores (e.g., carnivorans and cetaceans) and herbivores (e.g., ungulates). These carnivorous and herbivorous lineages scatter the phylogeny of Laurasiatheria, suggesting the occurrence of dietary transitions among them, while the evolutionary history of their diets remains less clear. For instance, living bats contain both carnivores (e.g., insect-eaters) and herbivores (e.g., fruit-eaters), and the diet of ancestral bats is still unknown, with both insectivory and frugivory having been proposed [1]. Likewise, for typical herbivores, such as odd-toed ungulates and even-toed ungulates, they are deeply nested within several carnivorous lineages, including carnivorans, pangolins, bats, and Eulipotyphla; however, another carnivore, the cetacean, is deeply nested within even-toed ungulates. This may suggest that their diets must have changed; however, the evolutionary history of their diets remains largely unknown, and few relevant studies exist. One previous macroecological study infers the evolutionary history of the diets within mammals, including Laurasiatheria, and a high frequency of their dietary transitions from herbivory and carnivory to omnivory is reported [2], which is a pattern found in birds as well [3]. More studies, especially at the molecular level, may be needed as reconstructing ancestral diets is of importance to understanding the evolutionary origin of the dietarily specialized taxa.

The recent development of a molecular phyloecological (MPE) approach provides a new opportunity to investigate ancestral traits using molecular data [4–7]. The MPE approach mainly uses the adaptive evolutionary analyses of the molecular markers indicative of trait states to determine the molecular basis of phenotypic differentiation and to infer ancestral traits given a phylogeny, and it has been used to infer the diel activity patterns and diets of ancestral taxa [4–8]. Regarding diet reconstruction, the MPE approach employs digestive system-related genes as the molecular markers indicative of diets to infer ancestral diets. Accordingly, carnivores are characterized by the selection intensification of protein and fat utilization, while herbivores are normally characterized by the selection intensification of carbohydrates [7–10] because carnivore diets are high in proteins and fats and herbivore diets are normally high in carbohydrates [7, 9–13]. The MPE method has been used to infer ancestral diets in birds [7, 8], and its fitness to mammals remains to be explored. In this study, we used Laurasiatheria as an ideal clade to examine the molecular basis underlying the diet differentiations and to reconstruct their ancestral diets as it contains the taxa with highly specialized diets. Our results provide new insights into understanding their evolutionary history of diets.

## Results

**Fig. 1:**
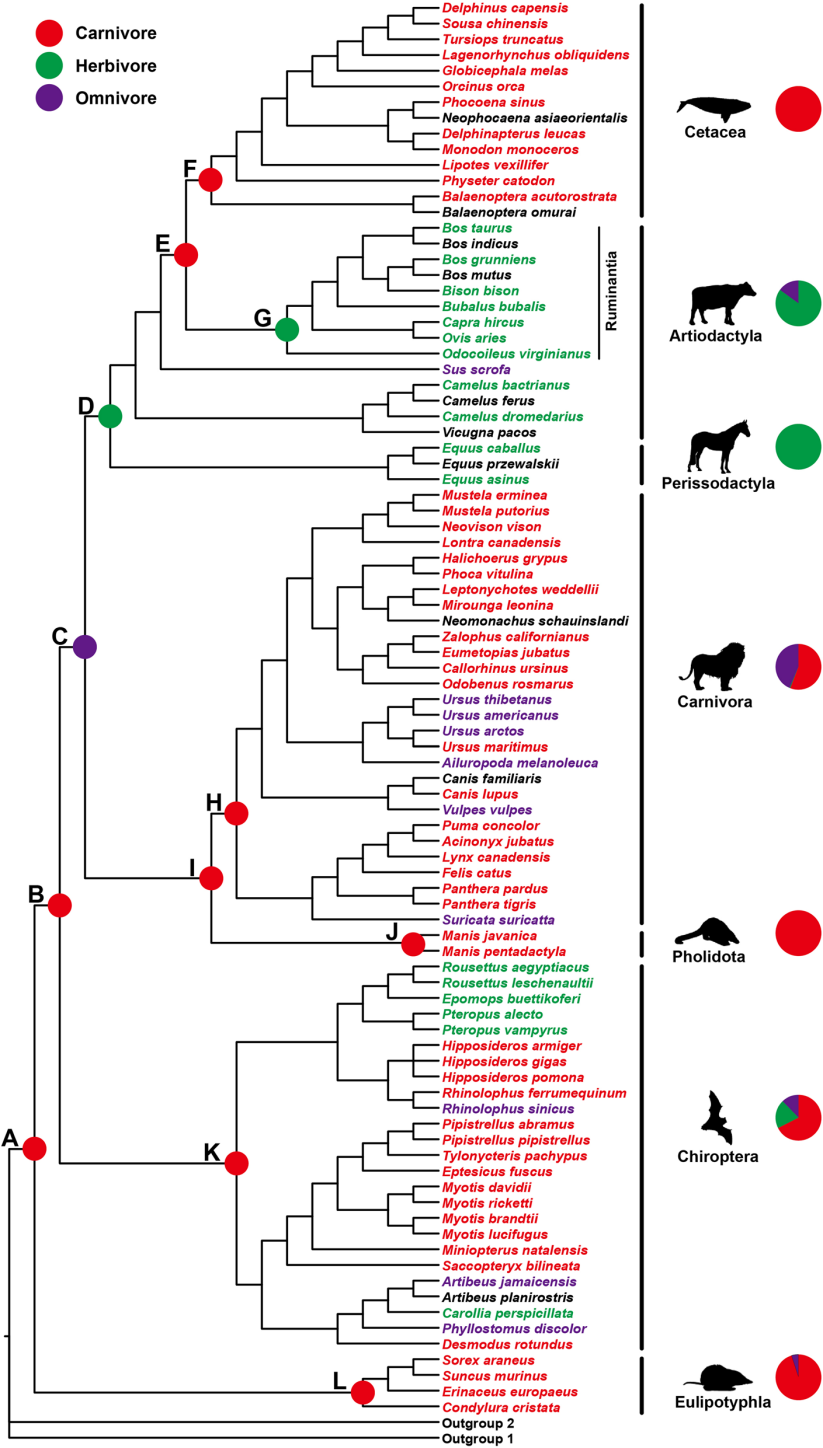
Laurasiatheria phylogeny and reconstructed ancestral dietary categories based on molecular data. The phylogenetic relationships among species follow published studies [94–96]. The branches under positive selection analyses are shown with letters (A–L). The dietary categories of each extant species and each mammalian order shown in the pet charts are based on one previous study [93]. Carnivores are shown in red, herbivores in green, and omnivores in violet. Black shows the species with no dietary categories available

**Fig. 2:**
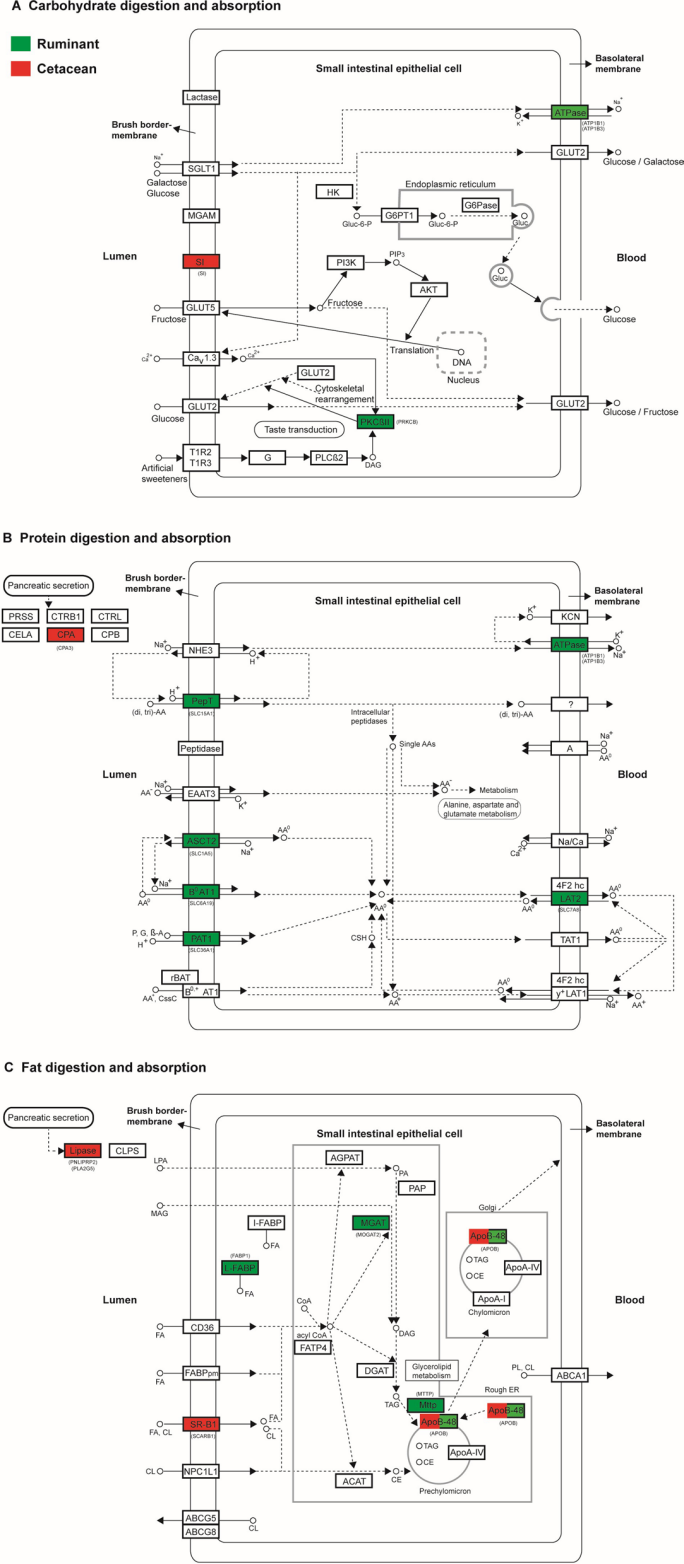
Digestive system pathways and positively selected genes found in ruminants (green) and cetaceans (red). Three digestive system pathways (**A**, **B**, and **C**) were modified from that of KEGG, including carbohydrate digestion and absorption (map04973), protein digestion and absorption (map04974), and fat digestion and absorption (map04975). Positively selected genes are shown in parentheses, and their corresponding proteins are highlighted in red (cetaceans) and green (ruminants)

Following the MPE approach to examine the molecular evolution of diets [7, 8], we examined the adaptive evolution of 119 digestive system-related genes (Additional file 2: Table S1) in the given Laurasiatheria phylogeny (Fig. 1). These genes are involved in three KEGG pathways, and play important roles in the digestion and absorption of carbohydrates, proteins, and fats [7] (Fig. 2). The positive selection of these genes along particular branches (A–L in Fig. 1) was analyzed using branch and branch-site models implemented in PAML software [14]. Positively selected genes (PSGs) were mainly detected based on the branch-site model (Additional file 3: Table S2). We initially analyzed the positive selection along the lineages with highly specialized diets, including three primarily carnivorous lineages (Eulipotyphla, Pholidota, and Cetacea) and one typically herbivorous lineage (Ruminantia). Intriguingly, our results reveal a highly similar selection pattern across the four lineages and show the predominant selection of fat and protein utilization with relatively the weakest selection of carbohydrate utilization in terms of both the p values and the number of PSGs found. These results remain unchanged even after the Bonferroni multiple testing correction of the p values of PSGs (Additional file 3: Table S2, Additional file 4: Table S3, Additional file 5: Table S4).

Eulipotyphla primarily eats invertebrate prey, and we found 10 PSGs along the branch leading to Eulipotyphla (branch L, Fig. 1), including four fat utilization-related genes (*APOB*, *APOA1*, *LIPF*, and *NPC1L1*), four protein utilization-related genes (*MEP1B*, *CTRL*, *SLC3A2*, and *CPA1*), and two carbohydrate utilization-related genes (*HK1* and *MGAM*) (Additional files 3, 4, 5: Tables S2-4). Among these PSGs, *APOB* and *APOA1* encode key apolipoproteins responsive to carrying fats and fat-like substances in the blood [15, 16]. *LIPF* encodes a gastric lipase, which plays an important role in the digestion of dietary triglycerides in the gastrointestinal tract [17]. *NPC1L1* is responsible for the intestinal absorption of cholesterol and/or plant sterols [18]. *MEP1B* encodes metalloendopeptidases [19]. *CTRL* is considered to play a role in the digestion of proteins [20]. *SLC3A2* encodes an amino acid transporter [21]. *CPA1* encodes a pancreatic exopeptidase [22]. *HK1* encodes hexokinase 1, which catalyzes the first step in glucose metabolism [23]. *MGAM* encodes maltase-glucoamylase, which is involved in the small intestinal digestion of starch to glucose [24].

Pholidota eats almost exclusively ants and termites. Our positive selection analyses along the Pholidota branch (branch J, Fig. 1) revealed 16 PSGs (Additional file 3: Table S2), including six fat utilization-related genes (*APOB*, *PLPP2*, *APOA1*, *SLC27A4*, *PLA2G1B*, and *CLPS*), six protein utilization-related genes (*SLC8A2*, *SLC7A9*, *SLC3A1*, *DPP4*, *KCNN4*, and *SLC3A2*), and four carbohydrate utilization-related genes (*MGAM2*, *HK3*, *G6PC*, and *LCT*). *PLPP2* functions in phospholipid metabolism by converting phosphatidic acid to diacylglycerol [25]. *SLC27A4* is known as an important fatty acid transporter in small intestinal enterocytes [26]. *PLA2G1B* encodes phospholipase A2 and catalyzes the release of fatty acids from glycero-3-phosphocholines [27]. *CLPS* encodes a pancreatic colipase [28]. *SLC8A2* encodes a Na^+^-Ca^2+^ exchanger, which is widely expressed in different tissues [29]. *SLC7A9* is involved in amino acid transport [30]. *SLC3A1* encodes an amino acid transporter [31]. *DPP4* codes a cell-surface protease [32]. *KCNN4* codes for the calcium-activated potassium channels [33]. *MGAM2* is involved in the degradation of starch or glycogen and is highly expressed in the small and large intestines [34]. *HK3* is involved in glucose metabolism [35]. *G6PC* plays an important role in the homeostasis regulation of blood glucose concentrations, catalyzing the terminal step in gluconeogenesis and glycogenolysis [36]. *LCT* encodes a molecule with both lactase activity and phlorizin hydrolase activity [37].

Cetaceans are primarily carnivores, feeding on invertebrates and vertebrate prey. Our positive selection analyses detected six PSGs along the ancestral branch of Cetacea (branch F, Fig. 1), including four fat utilization-related genes (*APOB*, *PNLIPRP2*, *PLA2G5*, and *SCARB1*), one protein utilization-related gene (*CPA3*), and one carbohydrate utilization-related gene (*SI*) (Fig. 2; Additional file 3: Table S2). *PNLIPRP2* is a pancreatic lipase-related protein [38]. *PLA2G5* is a member of the phospholipase A2 gene family and plays a role in the hydrolysis of phospholipids [39]. *SCARB1* mediates the uptake of cholesterol and a variety of lipids [40]. *CPA3* is involved in the degradation of proteins [41]. *SI* encodes sucrase-isomerase and is essential for the digestion of dietary carbohydrates including starch, sucrose, and isomaltose [42].

Ruminantia is typically herbivorous. Our positive selection analyses revealed 12 PSGs along the ancestral branch leading to Ruminantia (branch G, Fig. 1), including four fat utilization-related genes (*APOB*, *MOGAT2*, *MTTP*, and *FABP1*), five protein utilization-related genes (*SLC36A1*, *SLC6A19*, *SLC1A5*, *SLC7A8*, and *SLC15A1*), one carbohydrate utilization-related gene (*PRKCB*), and two ionic homeostasis-related genes (*ATP1B1* and *ATP1B3*) [43] involved in both protein and carbohydrate utilization (Fig. 2; Additional file 3: Table S2). *MOGAT2* plays a role in dietary fat absorption from the small intestine [44]. *MTTP* catalyzes the transport of triglycerides, cholesteryl esters, and phospholipids [45]. *FABP1* encodes a fatty acid-binding protein that regulates lipid transport and metabolism [46]. *SLC36A1*, *SLC6A19*, *SLC1A5*, and *SLC7A8* mediate the transport of amino acids [47–50]. *SLC15A1* encodes an intestinal transporter of peptides [51]. *PRKCB* encodes a protein kinase involved in many different cellular functions, including intestinal sugar absorption [52].

We also analyzed the positive selection of the digestive system-related genes in Chiroptera and Carnivora, both of which harbor dietary diverse species. Chiroptera contains both carnivores (e.g., insect-eaters) and herbivores (e.g., fruit-eaters), and our positive selection analyses along the ancestral Chiroptera branch (branch K, Fig. 1) revealed eight PSGs (Additional file 3: Table S2, Additional file 4: Table S3, Additional file 5: Table S4). These eight PSGs include only protein utilization-related genes (*SLC3A2*, *SLC1A5*, *CELA3B*, and *DPP4*) and fat utilization-related genes (*APOB*, *CD36*, *ABCG8*, and *PLPP2*). *CELA3B* is a pancreatic serine proteinase that digests dietary protein substrates [53]. *CD36* is mainly involved in the uptake and processing of fatty acids [54]. *ABCG8* functions in the excretion of neutral sterols in the liver and intestines [55]. Like Chiroptera, for the ancestral branch of Carnivora (branch H, Fig. 1), only fat utilization-related genes (*APOB* and *PIK3CD*) and protein utilization-related genes (*CPB2* and *KCNK5*) were found to be under positive selection (Additional file 3: Table S2). *PIK3CD* encodes phosphatidylinositol 3-kinase with a broad phosphoinositide lipid substrate specificity [56]. *CPB2* encodes carboxypeptidase B2, cleaving C-terminal residues from peptides [57]. *KCNK5* is considered to play an important role in potassium transport [58].

To determine the selection characterization of ancestral taxa, we subsequently examined the positive selection of the digestive system-related genes along other early branches of Laurasiatheria (branches A, B, C, D, E and I, Fig. 1) (Additional file 3: Table S2, Additional file 4: Table S3, Additional file 5: Table S4). For the ancestral Laurasiatheria branch (branch A, Fig. 1), we found two fat utilization-related genes (*APOB* and *NPC1L1*), one protein utilization-related gene (*MEP1B*), and one glucose metabolism-related gene (*HKDC1*) [59] to be under positive selection. For branch B, only one fat utilization-related gene, *AGPAT2*, was found to be under positive selection. It plays a role in converting lysophosphatidic acid into phosphatidic acid [60]. For branch C, three PSGs (*SLC7A8*, *MGAM2*, and *ATP1B3*) were found. For branch I, one positively selected fat utilization-related gene, *APOB*, was detected. For branch D, two fat utilization-related genes (*APOB* and *PLA2G2D*) were found to be under positive selection, and notably, *PLA2G2D* is a member of lipolytic enzyme [61]. For branch E, two fat utilization-related genes (*LIPF* and *PNLIP*) and two protein utilization-related genes (*SLC3A2* and *MEP1B*) were found to be under positive selection, of which *PNLIP* encodes a pancreatic lipase, also known as pancreatic triacylglycerol lipase. This pancreatic lipase hydrolyzes dietary triglycerides to free fatty acids and monoacylglycerols and is critical for the efficient digestion of dietary triglycerides in the intestines [62, 63].

**Fig. 3:**
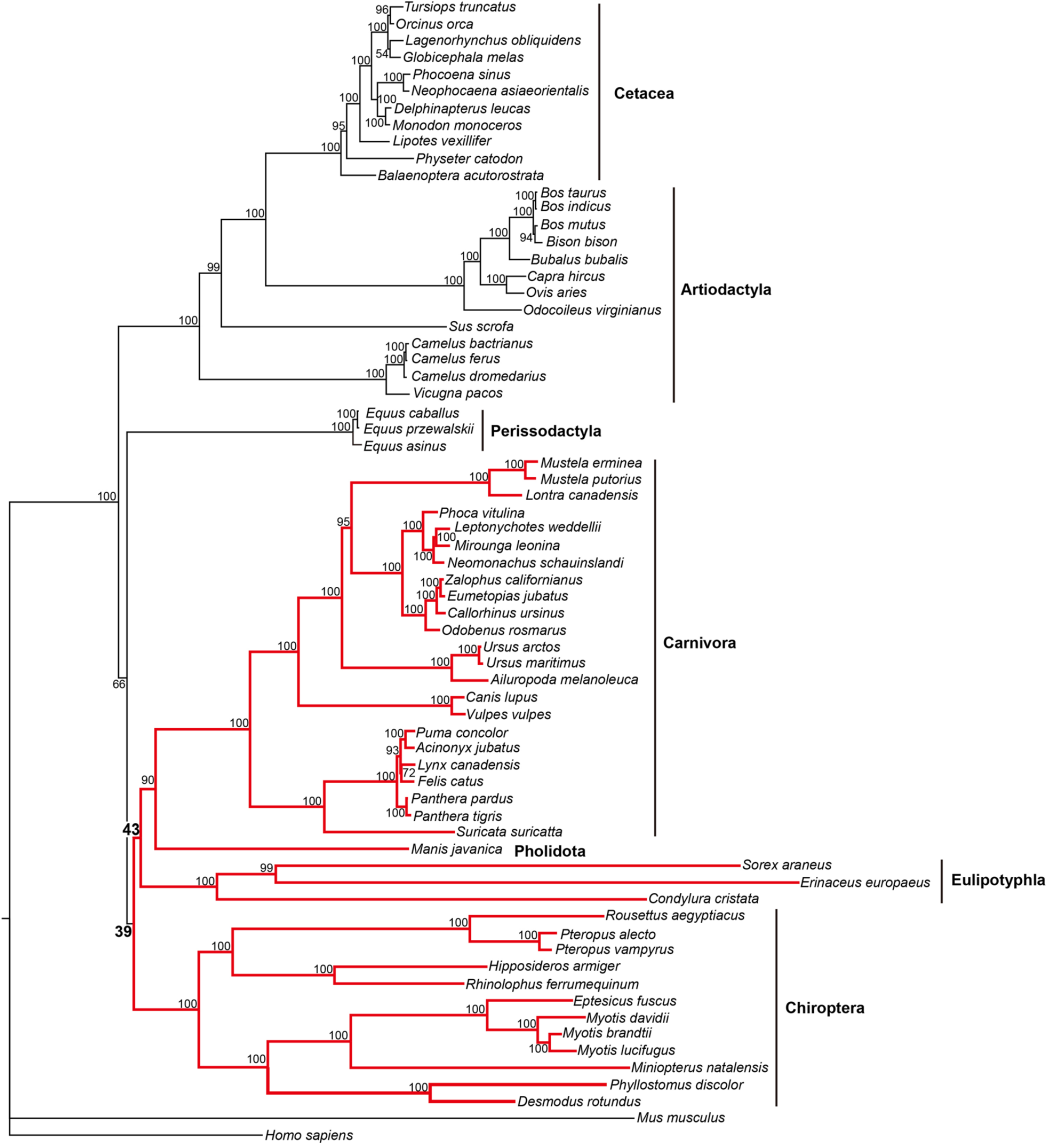
Maximum-likelihood phylogeny of the gene *APOB*. The phylogeny is based on 4550 amino-acid sites with the best-fit substitution model of the HIVb+F+R4 used. Red shows the clustering of four carnivory-dominant taxa

Among the PSGs found, one gene *APOB* showed a particularly strong positive selection with hundreds of positively selected amino acid sites found in most taxa examined, including those carnivorous taxa (Additional file 3: Table S2). To test whether the *APOB* gene was subject to convergent evolution among the carnivorous taxa, we subsequently examined the convergent and/or parallel amino acid substitutions along the branches related to those carnivores by reconstructing ancestral sequences using PAML [14], and many parallel amino acid substitutions were detected among them with high statistical significance (Additional file 1: Fig. S1; Additional file 6: Table S5). For instance, nine parallel substitutions each were found between the branches of Eulipotyphla and Pholidota, and between the branches of Chiroptera and Pholidota. Eight parallel substitutions were found between the branch of Carnivora and two other branches of Eulipotyphla and Chiroptera. These parallel amino acid substitutions may have led to their sequence convergence and thus to their phylogenetic affinity. To test this, we then constructed the maximum likelihood phylogeny based on the protein sequence of the gene *APOB*. Intriguingly, our results showed that the *APOB* tree (Fig. 3) was largely different from their species tree (Fig. 1). In particular, we found that four carnivory-dominant taxa (Carnivora, Pholidota, Chiroptera, and Eulipotyphla) were grouped into the same clade with bootstrap values ranging from 39 to 43 upon three independent runs, indicating their sequence convergence.

## Discussion

We in this study examined the adaptive evolution of digestive system-related genes to determine the diet evolution within Laurasiatheria. Consistent with previous studies that demonstrate the evolutionary enhancement of protein and fat utilization in carnivores [7, 9, 10], all three primarily carnivorous mammal taxa (Eulipotyphla, Pholidota, and Cetacea) examined in this study showed a particularly intensified selection of fat and protein utilization with relatively the weakest positive selection of carbohydrate utilization (Additional file 3: Table S2, Additional file 4: Table S3, Additional file 5: Table S4). This is consistent with their high-protein and high-fat diets. Unexpectedly, for the typical herbivores, the ruminants, which have a high-carbohydrate diet, we detected an intensified selection of fat and protein utilization rather than carbohydrate utilization (Additional file 3: Table S2), resembling that of carnivores. This may suggest that convergent evolution may occur between the carnivores and the herbivores studied. The convergent evolution of diet-related genes is often considered to be resulted from the utilization of similar food [12, 64–66], while its occurrence in the ruminants, as evidenced previously [67–69], may largely attribute to their specialized digestive system rather than similar food. As we know, ruminants primarily consume plant materials rich in carbohydrates, but they have no enzymes to digest the refractory materials (e.g., cellulose) in their diets. These refractory materials are transferred through microbial fermentation in their guts to volatile fatty acids and microbe proteins, constituting the major sources of energy and amino acids for subsequent utilization by ruminants [70–73]. This suggests that though their diets are rich in carbohydrates, the main nutritional substrates that ruminants are capable of directly utilizing are actually fats and proteins that are generated through microbial fermentation. Therefore, the intensified selection of fat and protein utilization found in the ruminants may be mainly due to their specialized digestive system.

Our results show that the carnivorous mammals studied are consistently characterized by an intensified selection of fat and protein utilization, which is in line with their high-protein and high-fat diets. For herbivorous mammals, because fermentation through their gut microbes can transfer dietary carbohydrates to other nutritional substrates, such as volatile fatty acids and microbial proteins, for their subsequent use [70–73], the selection characterization of their digestive system-related genes do not necessarily reflect the amounts of nutritional substrates in their diets. Previous studies suggest that microbial fermentation widely occurs in animals, while the contribution of microbial fermentation to energy production is largely different among animals. Its importance seems to be limited to particular taxa (e.g., ungulates), possibly due to high amounts of refractory materials (e.g., cellulose) in their diets, but is relatively trivial to other animals [74]. Consequently, the adaptive evolution of digestive system–related genes of animals is considered to be generally positively related to loads of their dietary substrates [10, 12, 13, 74–76]. Accordingly, carnivores are characterized by the selection intensification of protein and fat utilization, while herbivores are normally characterized by the selection intensification of carbohydrates [7, 9, 10]. Thus, we could reconstruct the diets of ancestral taxa based on the selection characterization of digestive system-related genes [7, 8].

To determine ancestral diets, we analyzed the positive selection of the digestive system-related genes along the ancestral branches of the living animals studied (Fig. 1). For the ancestral branches of bats (branch K) and of carnivorans (branch H), we detected their evolutionary enhancements mainly in fat and protein utilization, as found in the ancestral branches leading to other primarily carnivorous mammals (Eulipotyphla, Pholidota, and Cetacea) (Additional file 3: Table S2, Additional file 4: Table S3, Additional file 5: Table S4). This may suggest that all the ancestral taxa including ancestral bats and ancestral carnivorans were largely carnivorous (Fig. 1). Similarly, an evolutionary enhancement of fat and/or protein utilization was also found in other ancestral branches (branches A, B, and I) (Additional file 3: Table S2). This may suggest that the early evolutionary diversification of Laurasiatheria was mainly characterized by carnivory (Fig. 1), which is largely consistent with one previous study [2]. Nonetheless, for the ungulates examined in this study, we unexpectedly found an intensified selection of fat utilization along branch D, leading to ancestral ungulates, and an evolutionary enhancement of fat and protein utilization along branch G, leading to ruminants (Additional file 3: Table S2). This may reflect their high fat and/or protein nutrition generated by nutritional transformation through microbial fermentation, and hence it may suggest that the ancestral ungulate and the ancestral ruminant may be herbivorous (Fig. 1). For branch C, we detected the positive selection of three PSGs involved in carbohydrate and protein utilization (Additional file 3: Table S2, Additional file 4: Table S3, Additional file 5: Table S4), suggesting a high-carbohydrate and high-protein diet. A high-carbohydrate and high-protein diet may suggest a combination of herbivory and carnivory, hence implying that the common ancestor of carnivorans and ungulates was possibly omnivorous (Fig. 1), which may be derived from its carnivorous progenitor (branch B). If this is the case, it may suggest that the herbivory of ungulates and the carnivory of carnivorans were secondarily evolved. This is consistent with fossil evidence showing that the earliest stem carnivorans, such as *Ravenictis* and *Pristinictis*, exhibit relatively unspecialized molars, indicating an omnivorous diet with only limited specialization to true carnivory [77]. Fossil evidence indicates the resemblance of some primitive ungulates to carnivores. For instance, *Phenacodus*, which is considered a stem Perissodactyla [78], lived during late Palaeocene and early Eocene, and was a plant-eater yet shows some characteristics (e.g., large canine teeth) resembling a primitive carnivore [79]. These lines of evidence may suggest the herbivory of the ungulates, and the pure carnivory found in modern carnivorans may be secondarily evolved from an omnivorous ancestor.

For the branch leading to the common ancestor of ruminants and cetaceans (CARC), we detected the enhanced selection of fat and protein utilization (Additional file 3: Table S2), which is similar to that found in both of its two derived taxa, the ruminants and the cetaceans. This seems to make the reconstruction of the diet of the CARC unresolved; however, our finding of the positive selection of the two fat digestion-related genes (*PNLIP* and *LIPF*) along the branch leading to the CARC (Additional file 3: Table S2) may suggest that the CARC was more likely carnivorous (Fig. 1). This is because: i) *PNLIP* and *LIPF* are both critical lipases mainly involved in digesting dietary triglycerides in the digestive system [17, 62, 63, 80], and the selection enhancement of the digestion of dietary triglycerides may suggest a lipid-rich diet of the CARC. A lipid-rich diet most often characterizes carnivores rather than herbivores because carnivore diets are relatively rich in lipids, while herbivore diets are normally rich in carbohydrates [9–13]. ii) The evolutionary enhancement of digesting dietary triglycerides found in the CARC may suggest that the CARC itself may have the capability to digest dietary fats (e.g., triglycerides). This is consistent with carnivorous mammals (e.g., cetaceans), which normally use their own lipases to digest fats [81], but is substantially different from ruminants, from which their dietary lipids (e.g., triglycerides) are predominantly hydrolyzed by the lipases of rumen bacteria in their guts [82–85]. iii) The detected positive selection of *PNLIP* and *LIPF* in the CARC has been found in carnivores (e.g., cetaceans) [68, 76] but not in ruminants, which is evidenced in this study and one previous study [68], suggesting the resemblance of the digestion ability of the CARC with that of carnivores (cetaceans). In addition to the molecular evidence, fossil evidence shows that early ruminant ancestors were omnivores and did not ruminate until about 40 Ma based on dental morphology [86]. This indicates that the herbivory and rumination observed in modern ruminants may be secondarily evolved, which is consistent with the possible carnivory of the CARC. These four lines of evidence may suggest that the CARC was more likely a carnivore, though its existence in fossils remains to be explored. Considering that the CARC is phylogenetically deeply nested within the ungulates, it is thus more likely a carnivorous ungulate closely related to cetaceans and/or ruminants. Among carnivorous mammals known, previous studies have long considered one extinct carnivorous ungulate, mesonychians, as early members of cetaceans or Cetartiodactyla [87–91], though there is uncertainty regarding the phylogenetic position of mesonychians [77, 92]. Mesonychians are considered secondary carnivores derived from archaic ungulates (Condylarthra) [88]. If this is the case, mesonychians might be the candidate of the CARC from which cetaceans and ruminants derived, though this requires further investigation.

## Conclusions

Our molecular phyloecological results show that the carnivorous mammals consistently exhibit the evolutionary enhancement of fat and protein utilization, which is in line with their high-protein and high-fat diets. This is previously found in birds and crabs as well. For herbivores, previous studies on birds and crabs suggest that they tend to show an evolutionary enhancement of carbohydrates; however, the ungulates with a high-carbohydrate diet examined in this study present an evolutionary enhancement of fat and protein utilization, resembling that of carnivores. Apparently, this is largely due to their specialized digestive system that transfers abundant carbohydrates to volatile fatty acids and microbial proteins for their use. Our results suggest that the adaptive evolution of digestive system-related genes do not necessarily reflect the nutritional loads in the diets of the herbivorous animals (e.g., ungulates) that mainly rely on nutritional transformation before utilization but appear to be positively related to the loads of the nutrient substrates that can be directly utilized by the herbivores themselves. Based on these findings, we reconstructed ancestral diets, and our results revealed predominant carnivory during the early diversification of Laurasiatheria. More importantly, our reconstructed results suggest that the ungulates and carnivorans may have been derived from an omnivorous ancestor, and ancestral bats and the common ancestor of ruminants and cetaceans may be carnivorous. We also found evidence of the convergent evolution of one fat utilization-related gene, *APOB*, across carnivorous lineages, suggesting the resemblance of nutritional utilization in carnivorous mammals. Further studies incorporating information about the gene duplications and losses besides positive selection may be helpful to understand the molecular bases underlying the diet evolution of the herbivorous and carnivorous mammals.

## Materials and methods

### Taxa used

Ninety species within Laurasiatheria were included (Fig. 1). These 90 species covered all known main clades of Laurasiatheria, including four species of Eulipotyphla, two pangolin species of Pholidota, three species of odd-toed ungulates (Perissodactyla), and 14 species of even-toed ungulates (Artiodactyla), of which nine species belong to Ruminantia. We also included 25 bat species from both two suborders (Yangochiroptera and Yinpterochiroptera) of Chiroptera. For Carnivora, 28 species from its two suborders (Feliformia and Caniformia) were included. For Cetacea, 14 species from its two suborders (Mysticeti and Odontoceti) were included. In addition to these 90 Laurasiatheria species, we included two species from the sister taxa (Euarchontoglires) of Laurasiatheria as outgroups. For the two outgroup species, *Homo sapiens* and *Mus musculus* were primarily used, while the two relatives (*Rattus rattus* and *Rattus norvegicus*) of *Mus musculus* were also considered if some gene sequences of *Mus musculus* were unavailable.

### Diet data

The dietary categories of each species used in this study were based on one previously published dataset, EltonTraits 1.0 [93], in which the dietary information of a total of 5400 extant mammal species from diverse published literature is compiled and the dietary composition of each species is recorded in 10% dietary categories. To determine the dietary categories of the species used in our study, we converted EltonTraits’ 10% dietary categories into our three dietary categories (carnivore, herbivore, and omnivore). Carnivore = Diet-Inv + Diet-Vend + Diet-Vect + Diet-Vfish + Diet-Vunk + Diet-Scav, herbivore = Diet-Fruit + Diet-Nect + Diet-Seed + Diet-PlantO, and omnivores were referred to as the animals that contain a percentage of dietary categories of both the carnivore and the herbivore.

### Genes and sequence alignment

We included the digestive system-related genes that have been recently used to determine the diet evolution in birds [7]. These genes were from three KEGG pathways, including carbohydrate digestion and absorption (map04973), protein digestion and absorption (map04974), and fat digestion and absorption (map04975). For these genes, we downloaded their coding sequences of our focal species from GenBank (Additional file 2: Table S1). We excluded genes with sequences unavailable or available for only a few species from our analyses, and ultimately, 119 genes were retained for subsequent analyses. We aligned gene sequences using webPRANK with default parameters (http://www.ebi.ac.uk/goldman-srv/webprank/), and individual species sequences with lengths that were too short were removed. The sequence alignments were checked by eye and the sequence gaps that lead to incorrect protein translation were cut. After this pruning, we blasted the translated protein sequences of these genes against the non-redundant protein sequence database to confirm the correctness of the sequence cutting.

### Positive selection analyses

For the positive selection analyses, we initially constructed a Laurasiatheria phylogeny of the 90 species used in this study, as shown in Fig. 1. Our Laurasiatheria phylogeny was based on published studies [94–96]. In particular, the phylogenetic relationships used among taxonomical orders within Laurasiatheria, which have received increasing support for the past 20 years [95–98], are the same as those used in one previous diet study of mammals [2]. Based on the Laurasiatheria phylogeny, we analyzed the positive selection of our target genes using branch and branch-site models implemented in the Codeml program of PAML [14]. The ratio of non-synonymous to synonymous substitutions per site (dN/dS or ω) was evaluated, and likelihood ratio tests (LRT) were employed to determine the statistical significance. Positive selection is determined by the value of ω > 1 with statistical significance. The Bonferroni multiple testing correction was used to adjust the p values.

*Branch model* Branch model allows for the variation of the ω ratio among branches in a given phylogeny, and it is used to detect the positive selection of genes on a particular branch. For the branch model analyses, we used a two-rate branch model by labeling our focal branches as foreground branches and the others as background branches. During the analyses, the goodness of fit of the two-rate branch model relative to the null model—that is one-rate branch model— was analyzed using the LRT. When a statistically significant value of ω > 1 was found in our foreground branches, to determine whether the value of ω > 1 of the foreground branch was further supported with statistical significance, we then compared the two-ratio branch model with the two-ratio branch model with ω = 1 fixed in our foreground branches.

*Branch-site model* The branch-site model allows for the variation of ω among sites in the protein and across phylogenetic branches, and it is used to detect positive selection affecting some sites along a particular branch. For the branch-site model analyses, we employed a branch-site test of positive selection (Test 2), which compares a modified model A with its corresponding null model with ω = 1 fixed. The modified model A assumes four classes of sites, and site class 0 and site class 1, respectively, represent evolutionarily conserved (0 < ω_0_ < 1) and neutral codons (ω_1_ = 1) for both background and foreground branches. Site classes 2a and 2b, respectively, represent evolutionarily conserved (0 < ω_0_ < 1) and neutral (ω_1_ = 1) codons for background branches, but allowed to be under positive selection (ω_2_ > 1) for the foreground branches. The goodness of fit of the modified model A was evaluated using the LRT by comparing it with a null model with ω = 1 fixed. Positively selected sites were identified by employing an empirical Bayes method.

### Ancestral sequence reconstruction and convergent evolution analyses

Amino acid-based marginal reconstruction implemented in the empirical Bayes approach in PAML [14] was used to reconstruct the ancestral sequence. For the marginal reconstruction, we employed two different substitution models (JTT and Poisson) of amino acids to examine the consistency of our results. For the model JTT, different substitution rates of different amino acids were assumed, and for the Poisson model, the same substitution rate of all amino acids was assumed. The analyses based on the two substitution models generated similar results, and for convenience, we only showed the results based on the JTT model. Based on the reconstructed ancestral sequences of internal nodes, convergent and/or parallel amino acid substitutions along branches could then be identified. To further estimate the probabilities that the observed convergent and/or parallel substitutions are attributable to random chance, the program converg2 implemented in the software Convergent and Parallel Evolution at the Amino Acid Sequence Level (CAPE) [99] was used.

### Phylogenetic analyses

Phylogenetic analyses were conducted using the IQ-TREE, a fast and effective stochastic algorithm for inferring maximum-likelihood (ML) phylogeny [100, 101]. The IQ-TREE is considered to have high performance for ML inference compared to other popular software, such as RAxML [102] and PhyML [103]. This is considered to result from its efficient integration of fast model selection, an effective tree search algorithm, and a novel ultrafast bootstrap approximation [100]. Especially, the effective tree search algorithm was believed to overcome the problem of local optima and thus to help to achieve ML phylogeny with higher likelihoods than RAxML or PhyML. For our phylogenetic analyses, 4550 amino-acid sites of the gene *APOB* were used. Among 546 protein models examined by ModelFinder implemented in the IQ-TREE, two models, HIVb+F+R4 and HIVb+F+R5, were recommended as the best-fit substitution models according to Bayesian and Akaike information criteria, respectively. For result robustness, the two substitution models were both used and almost identical results were obtained. For convenience, only the ML phylogeny based on the substitution model of the HIVb+F+R4 was presented with the bootstrap value of 10,000 used.

## Supplementary Information

**Additional file 1: Fig. S1.** Amino acid substitutions along carnivorous lineages.

**Additional file 2: Table S1.** Gene sequences with GenBank accession numbers used in this study.

**Additional file 3: Table S2.** Positively selected genes identified by branch-site model. P values were corrected by multiply them by the number of branches tested of each gene (Bonferroni multiple testing correction).

**Additional file 4: Table S3.** Positively selected genes identified by branch model.

**Additional file 5: Table S4.** Positively selected genes identified by branch model.

**Additional file 6: Table S5.** Tests of parallel evolution of the gene *APOB* between branches using software CAPE.

## Data Availability

The sequence alignment and tree file of each gene used in this study are deposited in the Dryad repository (doi:10.5061/dryad.3tx95x6gz).

## Abbreviations

CAPE: Convergent and parallel evolution at the amino acid sequence level
CARC: Common ancestor of ruminants and cetaceans
JTT: Jones–Taylor–Thornton
KEGG: Kyoto Encyclopedia of Genes and Genomes
LRT: Likelihood ratio tests
ML: Maximum-likelihood
MPE: Molecular phyloecological
PAML: Phylogenetic analysis by maximum likelihood
PhyML: Phylogenetic estimation using maximum likelihood
PSGs: Positively selected genes
RAxML: Randomized axelerated maximum likelihood
