# Supplementary figures and images for: Diet evolution of carnivorous and herbivorous mammals in Laurasiatheria
Source: BMC Ecol Evol. 2022 Jun 21;22:82. doi: 10.1186/s12862-022-02033-6 (PMC9210794; doi:10.1186/s12862-022-02033-6)

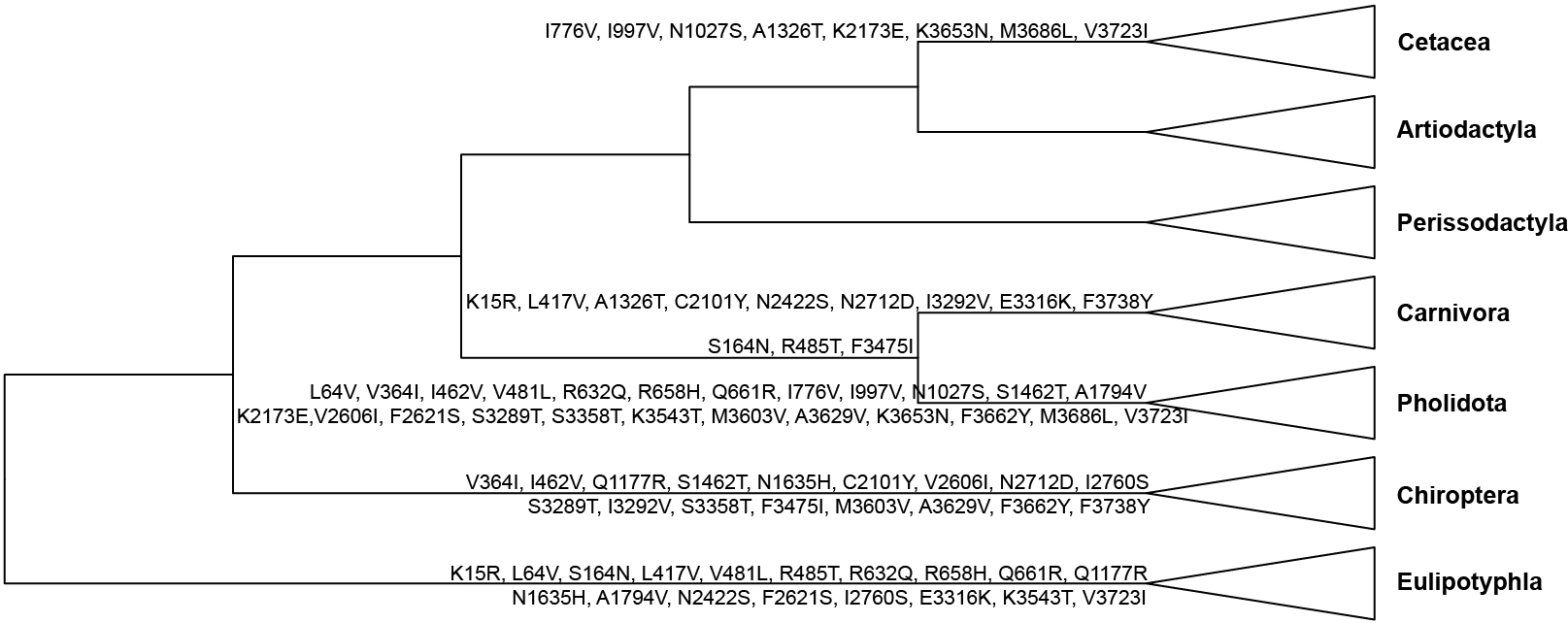

Supplement: Supplementary file 1 — Additional file 1: Fig. S1. Amino acid substitutions along carnivorous lineages. [file 12862_2022_2033_MOESM1_ESM.jpg]
